# Microstructure Imaging of Crossing (MIX) White Matter Fibers from diffusion MRI

**DOI:** 10.1038/srep38927

**Published:** 2016-12-16

**Authors:** Hamza Farooq, Junqian Xu, Jung Who Nam, Daniel F. Keefe, Essa Yacoub, Tryphon Georgiou, Christophe Lenglet

**Affiliations:** 1 Department of Electrical and Computer Engineering, University of Minnesota, Minneapolis, MN, USA; 2 Department of Radiology, Icahn School of Medicine at Mount Sinai, New York, NY, USA; 3 Department of Computer Science and Engineering, University of Minnesota, Minneapolis, MN, USA; 4 Center for Magnetic Resonance Research, Department of Radiology, University of Minnesota, Minneapolis, MN, USA.; 5 Department of Mechanical and Aerospace Engineering, University of California, Irvine, CA, USA.

## Abstract

Diffusion MRI (dMRI) reveals microstructural features of the brain white matter by quantifying the anisotropic diffusion of water molecules within axonal bundles. Yet, identifying features such as axonal orientation dispersion, density, diameter, etc., in complex white matter fiber configurations (e.g. crossings) has proved challenging. Besides optimized data acquisition and advanced biophysical models, computational procedures to fit such models to the data are critical. However, these procedures have been largely overlooked by the dMRI microstructure community and new, more versatile, approaches are needed to solve complex biophysical model fitting problems. Existing methods are limited to models assuming single fiber orientation, relevant to limited brain areas like the corpus callosum, or multiple orientations but without the ability to extract detailed microstructural features. Here, we introduce a new and versatile optimization technique (MIX), which enables microstructure imaging of crossing white matter fibers. We provide a MATLAB implementation of MIX, and demonstrate its applicability to general microstructure models in fiber crossings using synthetic as well as *ex-vivo* and *in-vivo* brain data.

This work presents a method to characterize tissue microstructure in the brain white matter from Diffusion MRI (dMRI) data. The method, named Microstructure Imaging of Crossing (MIX) white matter fibers, is unique in that it allows estimating detailed axonal features in the presence of multiple fiber orientations. This is achieved by exploiting a separable structure in the relevant optimization/fitting problem, which enables the combination of stochastic search algorithms and gradient-based methods. Experiments are reported, that demonstrate the broad applicability of the method to existing microstructure models, as well as to extensions of these models to multiple fiber orientations. The experiments have been carried out using synthetic, *ex-vivo*, and *in-vivo* brain data. Results can be reproduced with a MATLAB implementation of MIX which has been made available (Code availability section).

Diffusion Tensor Imaging (DTI)^1^ is the most widely used technique applicable to dMRI. It assumes a *single compartment* and is unable to distinguish diffusion patterns in heterogeneous biological compartments within a voxel. It only provides *non-specific* markers such as fractional anisotropy (FA) and mean diffusivity (MD)^2^ that cannot resolve microstructure features^3^. During the past decade, a variety of multi-compartment models have been proposed, that aim to capture more accurately the variability of diffusion in sub-voxel regions, such as intra-axonal and extra-axonal compartments. In particular, these models directly provide information about the white matter microstructure^4^,^5^. They include ball-and-stick^6^,^7^, CHARMED^8^,^9^ (composite hindered and restricted model of diffusion), AxCaliber^10^,^11^, ActiveAx^12^,^13^ or the Minimal Model of White Matter Diffusion (MMWMD), NODDI^14^ (Neurite Orientation Dispersion and Density Index) and DIAMOND^15^. The CONNECT^16^ project recently leveraged the CHARMED, AxCaliber and ActiveAx models, in combination with tractography methods, to improve structural connectivity mapping methods.

Recent “taxonomy” studies have extensively compared existing multi-compartment analytical models using *ex-vivo* rat brain^17^ and *in-vivo* human brain^18^ dMRI data, and provided a ranking using the Bayesian information criterion (BIC). They rank three-compartment (intra-axonal, extra-axonal and isotropic) models consistently higher than models with fewer compartments. We note however that these studies only consider models that are based on single fiber orientation, although 60 to 90% of the brain white matter have fiber crossings^19^ (i.e., have several fiber bundles with different orientation in each voxel). The apparent obstacle in considering additional compartments and multiple fiber orientations can be traced back to the complex mathematical nature of the model functions and required fitting techniques.

Parameter estimation in multi-compartment models requires non-convex optimization even for a single axonal orientation. The task is further complicated by (i) the type of nonlinear relations (Supplementary Note 1) between model parameters and dMRI data that render the problem ill-posed, and by (ii) the effect of noise, particularly in clinical (shorter) scans, which is significant.

Several methods and software packages have recently been published to estimate microstructural features from dMRI. Here, we focus on three toolkits which are specific to fitting complex (three-compartment) microstructure models: CAMINO^20^ (Open-Source Diffusion-MRI Reconstruction and Processing), AMICO^21^ (Accelerated Microstructure Imaging via Convex Optimization) and, NODDI^14^. Other toolkits include e.g. DSI Studio (http://dsi-studio.labsolver.org/), which implements the Restricted Diffusion Imaging (RDI)^22^ method and CHARMED^8^,^9^ (http://neuroimaging.tau.ac.il/ya/charmed.html). In CAMINO, AMICO and NODDI, fiber orientation is obtained using Diffusion Tensor (DT) estimation. The remaining parameters (specifying volume fractions, radius indices, density indices, orientation dispersion, etc.) are determined using nonlinear regression. The limitation of CAMINO, AMICO and NODDI in their ability to scale and handle multiple fiber orientations with complex biophysical models can be attributed to the fact they do not exploit the separable structure of the problem.

The need to reliably identify multiple fiber orientations per voxel, and associated microstructural parameters, has motivated recent attempts that are based on simple biophysical models^23^,^24^,^25^. For instance, AMICOx^23^ (AMICO in fiber crossing) estimates axon diameter indices in two fiber orientations (synthetic data only, using ActiveAx model in two orientations^26^). However, the approach has not yet been shown to scale so as to incorporate additional fiber orientations, generic tissue models, and real dMRI data. A second approach^24^ introduced the “spherical mean technique”, capable of factoring out the effects of fiber crossing to estimate “per-axon” parallel and perpendicular effective diffusion coefficients, and subsequently extract fiber orientation using spherical deconvolution. Similarly, estimation of NODDI in two directions^25^ for tractography uses fiber orientation estimates from neighboring voxels. Despite these efforts, which highlight the critical need for novel computational techniques, a method to fit complex biophysical models to dMRI data (e.g. with ten or more parameters) would significantly improve our ability to quantify intrinsic white matter properties of the human brain.

We introduce a novel regression method, which is robust and versatile. It enables to fit existing biophysical models with improved accuracy, and open the possibility to propose and test new models that were impossible to solve until now. It utilizes the Variable Separation Method^27^ (VSM) to distinguish parameters that enter in both, linear and non-linear manner, in the model (Methods). The estimation of non-linear parameters is a non-convex problem and is handled first. This is done by stochastic search that utilizes Genetic Algorithms (GA); GAs have been shown to be effective in approximating exponential time series models^28^. The task to estimate linear parameters amounts to a convex problem and can be solved using standard least squares techniques. These parameter estimates provide a starting point for a Trust Region method in search for a refined solution. A detailed description of the algorithm is given in the Methods section.

Specific attributes of the new method (MIX) are: (i) ability to estimate generic model parameters with multiple fiber orientations, (ii) ease in initializing the parameter search, and (iii) ability to cope with complex/realistic models that may have more than four compartments (an apparent limit for all existing methods). With MIX, it is only the amount of available dMRI data that may impact the variance of parameter estimates and, thereby, indirectly limit the achievable number of compartments.

## Results

### Synthetic data experiments

We have evaluated MIX on synthetic data and compared the results with the CAMINO, AMICO and NODDI toolboxes. Four different tissue models were considered and are described in Supplementary Note 2 (with details on specific compartment model functions and dMRI signal provided in Supplementary Note 1). Not all of these methods support all four tissue models, with the exception of MIX. We follow the model naming convention used in the dMRI microstructure literature^17^,^18^,^21^. These models and associated parameters are:

- ActiveAx^12^,^13^ (i.e. Zeppelin-Cylinder-Ball-Dot): Volume fractions, axon radius index, perpendicular diffusivity and fiber orientation.
- NODDI^14^: Volume fractions, orientation dispersion, perpendicular diffusivity and fiber orientation.
- Tensor-Stick-Dot: Volume fractions, perpendicular and parallel diffusivities and fiber orientation.
- Zeppelin-Cylinder-Dot (ZCD) with three orientations (ZCDx), which is an extension of ZCD to multiple orientations^26^ and is introduced herein in order to demonstrate the ability of MIX to estimate complex axonal parameters in crossing fibers configurations. ZCDx’s parameters are similar to ActiveAx for each of the three possible fiber orientations (i.e. each orientation has its own volume fractions, radius index, diffusivity and orientation).

We generated synthetic data with known parameters for the four tissue models (Supplementary Note 2) using the CAMINO and NODDI toolboxes.

#### ActiveAx and NODDI

We then used MIX to estimate the parameters for the ActiveAx and NODDI models. Although these models “only” have respectively eight and six unknown parameters, we find that the absolute error between estimated and actual parameters is significantly lower for MIX, for SNR values of 1000, 20 and 8, as compared to CAMINO, AMICO and NODDI toolboxes (Supplementary Figs 1 and 2). In addition, we find that AMICO tends to over-estimate low axonal density values (below 0.01) with ActiveAx, and to provide limited contrast in radius values (which are limited to the range 4 to 6 microns). We hypothesize that this may be due to the required regularization in AMICO. MIX is not affected by such requirement and provides estimates that are closer to the ground truth and CAMINO’s estimates. For NODDI, we also note that estimation of orientation dispersion and intra-cellular volume fractions are particularly challenging for all methods, respectively for high dispersion and low volume fractions. MIX outperforms the other methods for orientation dispersion estimation while AMICO performs better for intra-cellular volume fractions.

#### Tensor-Stick-Dot

For the Tensor-Stick-Dot model, which has nine unknown parameters, we only compare MIX with CAMINO because AMICO has not yet been implemented for such a model. For this experiment, MIX was vastly superior to CAMINO, even when using 100 Levenberg-Marquardt runs, both in terms of accuracy as well as computational efficiency (Supplementary Fig. 3), when estimating perpendicular and parallel diffusivities.

#### ZCDx

Finally, for the ZCDx model, with nineteen parameters, MIX provided accurate and robust parameter estimates even at a reasonably low signal to noise ratio (SNR = 8). Since no other method is capable of estimating parameters for ZCDx, a comparison could not be carried out. However, this illustrates the ability of MIX to reliably estimate the parameters of a complex model, which might provide novel insight into the brain microstructure.

From a computational standpoint, throughout our experiments AMICO was the most efficient for ActiveAx and NODDI. However, for more complex biophysical models, AMICO has not yet been implemented, as it relies on search over a dictionary of nonlinear dependences, which appears prohibitively expensive. For problems amenable to CAMINO, MIX is in general twice as fast (Supplementary Note 3).

### Real data experiments

We evaluated MIX on real dMRI data, in four different experiments.

#### ActiveAx

**Figure 1:**
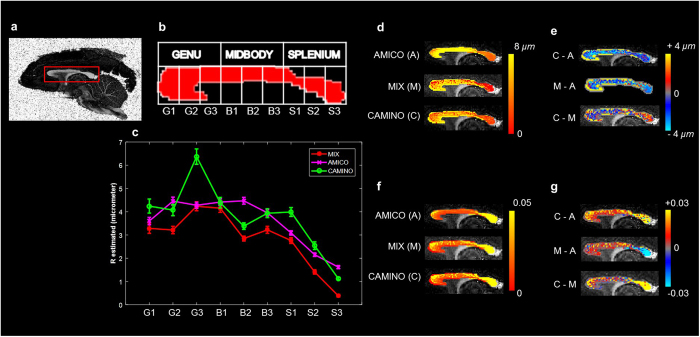
ActiveAx *ex-vivo* parameter estimation comparison using fixed monkey brain data. (**a**) Region of interest (in red box). (**b**) Partitions of the corpus callosum for this study. (**c**) Error plots (showing mean and standard deviation) for the axon radius index estimates in each partition. (**d**) Axon Diameter Index (D) estimates. (**e**) Differences between D estimates (**f**) Axon Density Index (DI) estimates. (**g**) Differences between DI estimates.

In the first experiment, we used *ex-vivo* monkey brain data^29^ from the original ActiveAx study^12^. Briefly, data (http://dig.drcmr.dk/activeax-dataset/) was obtained using a Varian 4.7 T system with voxel size 0.4 mm^3^, four *b*-values each with 90 directions and with the two lowest identical, and 12 additional *b* = 0 volumes. *b*-values and corresponding parameters are as follows: *b*_1_ = 1930 s.mm^−2^ (Δ/δ = 16/10 ms, |G| = 140 mT.m^−1^), *b*_2_ = *b*_1_, *b*_3_ = 3090 s.mm^−2^ (Δ/δ = 45/7 ms, |G| = 131 mT.m^−1^), *b*_4_ = 13190 s.mm^−2^ (Δ/δ = 35/17 ms, |G| = 140 mT.m^−1^)^29^. This experiment focused on fitting the ActiveAx model to a mid-sagittal slice of the corpus callosum (CC) using CAMINO, AMICO and MIX toolboxes. Axonal diameter index estimates show that all the algorithms exhibit similar patterns throughout the CC (Fig. 1d,f). More specifically, smaller axonal diameter indices and densely packed axons were identified in the *genu* and *splenium*, with larger axonal diameter indices and lower axonal densities in the mid-body. MIX, however, consistently provides smaller axonal diameter indices throughout the CC (Fig. 1c). This appears to be more realistic and in line with recent studies^30^ showing that axonal radii are always over-estimated using dMRI and existing scanning and fitting methods. The improvement appears to have been gained by our proposed data fitting method (MIX).

#### NODDI

**Figure 2:**
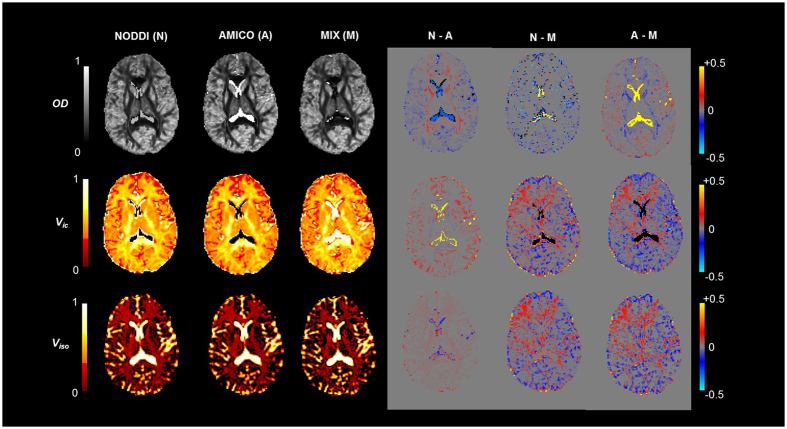
NODDI parameters estimation comparison for *in-vivo* human brain data. Fiber Orientation Dispersion (OD), intra-cellular volume fraction (*v*_*ic*_) and isotropic volume fraction (*v*_*iso*_) estimates are shown in first, second, and third row respectively. Left most column shows estimates by NODDI toolbox (N), second from the left by AMICO (A), third column from the left by MIX (M). The three columns on the right side show differences between the estimates of the three algorithms.

In the second experiment, we considered the NODDI model for *in-vivo* human brain data^14^ from the original NODDI study. The data was obtained on a 3T Philips Achieva system with voxel size 2 mm^3^ and two *b-*values of 711 s.mm^−2^ with 30 directions, and 2855 s.mm^−2^ with 60 directions. Nine *b* = 0 volumes are included in the protocol and Δ/δ are fixed to 37.8/17.5 ms. |G| is varied to achieve the desired *b-*values. We compared MIX with AMICO and NODDI and found that the three algorithms show similar trends for the estimated parameters in the white matter (Fig. 2). Largely, MIX and NODDI are in very close agreement with regard to fiber Orientation Dispersion index (OD), while MIX offers better contrast for intracellular volume fraction and isotropic volume fraction (Fig. 2, lower right four figures, see e.g. frontal white matter) as compared to the other two. In the cerebro-spinal fluid (CSF), MIX gives high values for intra-cellular volume fraction, whereas the other two predict the exact opposite. The discrepancy can be attributed to the fact that MIX explicitly constrains the sum of the volume fractions to be one. Therefore, estimates by MIX are relative to extracellular volume fraction only, and not to isotropic volume fraction (Supplementary Note 4). Nonetheless, estimated isotropic volume fraction is high in CSF areas, as expected, and in agreement with AMICO and NODDI.

#### Zeppelin-Cylinder-Cylinder-Dot (ZCCD)

In the third experiment, we used the ZCCD model (Supplementary Note 5) to demonstrate the performance of MIX in multiple axonal orientations. The choice of model is intended to allow improved modeling of complex tissue geometry of *in-vivo* human brain. Cylinders model water diffusion in two intra-axonal bundles (if they exist in a given voxel) and the zeppelin represents extra-axonal diffusion. Both the cylinders and zeppelin can have arbitrary orientations. Additionally, if the data does not support more than one or two fiber orientations, the corresponding volume fractions will be automatically driven to zero. Data was acquired on a healthy volunteer using a Siemens 3T Skyra system with voxel size 2 mm^3^, four *b*-values, each with 128 directions, and 9 additional *b* = 0 volumes. *b*-values and the corresponding parameters were chosen as follows: *b*_1_ = 820 s.mm^−2^ (Δ/δ = 17.6/9 ms, |G| = 98.5 mT.m^−1^), *b*_2_ = 980 s.mm^−2^ (Δ/δ = 55.5/5.2 ms, |G| = 97.1 mT.m^−1^), *b*_3_ = 3010 s.mm^−2^ (Δ/δ = 38.5/22.2 ms, |G| = 52.4 mT.m^−1^) and *b*_4_ = 7600 s.mm^−2^ (Δ/δ = 37.8/29.3 ms, |G| = 66.6 mT.m^−1^). The study was approved by the University of Minnesota Institutional Review Board and informed consent was obtained from the research participant. All methods were performed in accordance with the relevant guidelines and regulations.

**Figure 3:**
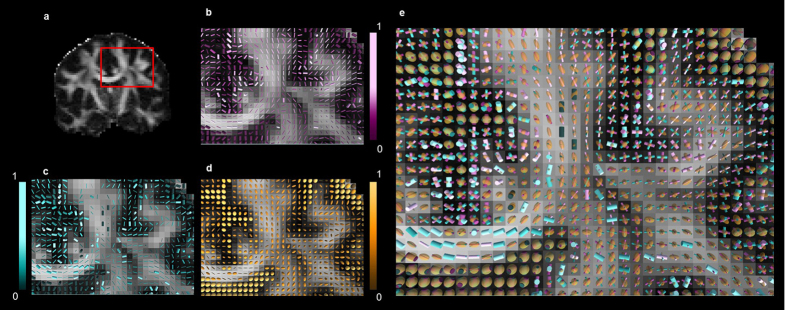
Zeppelin-Cylinder-Cylinder-Dot parameter estimation for *in-vivo* human brain data. (**a**) FA map for coronal slice and region of interest marked in red. (**b**) Cylinder 1. (**c**) Cylinder 2. (**d**) Zeppelin. (**e**) Zeppelin-Cylinder-Cylinder super-imposed. Color intensities show volume fractions for each compartment respectively. Both cylinders show estimated radii (μm) while zeppelin glyph thickness shows perpendicular diffusivity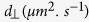.

Results show that in the corpus callosum (CC), both cylinders and zeppelin are aligned. In the area, one of the cylinders and the zeppelin have very small densities, therefore their associated axonal radii estimates and perpendicular diffusivity are meaningless and axonal radii estimates can be inferred from the cylinder with non-zero volume fraction (e.g. density). These findings conform to the well-known single dominant fiber orientation in the CC area (Fig. 3b–e). In contrast, in the centrum semi-ovale (CSO), where callosal, cortico-spinal and superior longitudinal fasciculus fibers cross, cylinders and zeppelin have distinct orientations and significant volume fractions, thereby reflecting the existence of multiple white matter pathways. We note that, for some voxels where these three major pathways are known to simultaneously exist and cross, the zeppelin tend to align with the third orientation. This is due to the fact that the ZCCD model only has two cylinders to model intra-axonal compartments and that, at these particular locations, the data more strongly supports a third intra-axonal, rather than the extra-axonal compartment. In the CSF, zeppelins have the highest volume fraction and large perpendicular diffusivity (Fig. 3d) while cylinders have very low volume fraction, capturing the largely free and isotropic diffusion occurring at this location. These findings can also be related to FA values (Fig. 3a) in CSF, which are very small since there is no dominant fiber orientation in this region. Although the results offer more comprehensive information (Fig. 3e), as compared to any single orientation biophysical model, further improvements in estimates accuracy could be made with model specific scanning protocol optimization.

#### NODDIx

**Figure 4:**
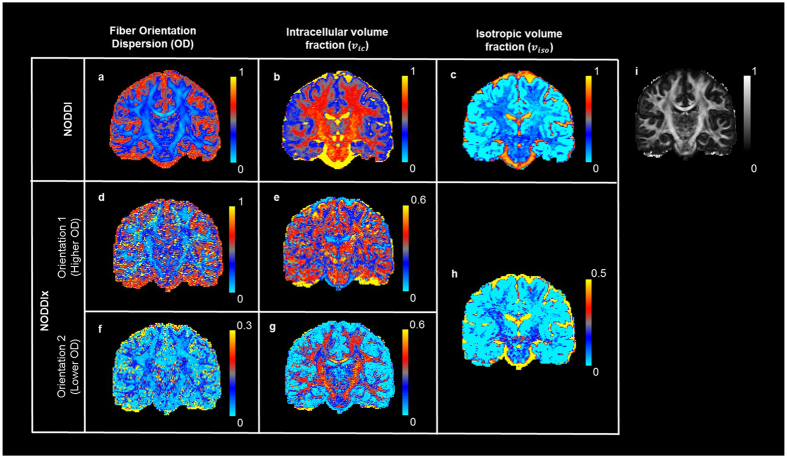
NODDI and NODDIx parameter estimation comparison using Human Connectome Project (HCP) data. First row shows results for NODDI model fitting while lower rows show results for NODDIx. Estimated model parameters in the two orientations for NODDIx have been sorted for OD values. It can be seen that *v*_*ic*_ for higher OD in (**e**) shows mostly grey matter while *v*_*ic*_ for lower OD in (**h**) shows white matter. (**a**) Fiber Orientation Dispersion (OD) (**b**) *v*_*ic*_ (**c**) *v*_*iso*_ (**d**) OD (higher values) along orientation 1. (**e**) *v*_*ic*_ for higher OD (orientation 1). (**f**) OD (lower values) along orientation 2. (**g**) *v*_*ic*_ for lower OD (orientation 2). (**h**) *v*_*iso*_. (**i**) FA map.

Finally, in the fourth experiment, we use our proposed ‘NODDIx’ model (Supplementary Note 6) to estimate *in-vivo* orientation dispersion (OD), intra-cellular (*v*_*ic*_) and isotropic (*v*_*iso*_) volume fractions in multiple fiber pathways. NODDIx is an extension of the NODDI model, which uses thirteen parameters to capture OD, *v*_*ic*_, *v*_*iso*_ and fiber orientations in up to two fiber pathways, and which can be accurately estimated with MIX. We use data from the Human Connectome Project (HCP)^31^ collected on a Siemens 3T Skyra system with voxel size 1.25 mm^3^, three b-values (1000, 2000, 3000 s.mm^−2^), each with 90 directions, and a total of 18 additional b = 0 volumes. Δ/δ are fixed to 43.1/10.6 ms, while |G| (Gmax = 97.4 mT.m-1) is varied to achieve the desired b-values. As shown in Fig. 4, NODDIx interestingly enables the differentiation of cortical/sub-cortical areas (i.e. mostly dendrites) or complex white matter areas like the centrum semi-ovale (i.e. crossings) with high orientation dispersion, from “simpler” white matter areas like the corpus callosum, cortico-spinal tract, etc. with low orientation dispersion. Contrary to NODDI, *v*_*ic*_ stays high in areas of high OD, presumably because the dMRI signal is better explained by the NODDIx model. This improved fit can also be concluded from the much lower residual values for *v*_*iso*_ throughout the brain, while still correctly identifying CSF outside the cortex and in the ventricles.

## Discussion

We have presented a technique (MIX) to fit complex biophysical models (e.g. ActiveAx, NODDI, NODDIx, ZCDx) to dMRI data of the brain, and estimate quantities such as axonal diameter and density. Unlike existing methods, MIX is versatile and thus suitable to a broad range of generic multi-compartment models, in particular for brain areas where axonal pathways cross. Since the prevalence of such complex brain areas is estimated to 60 to 90% of the white matter, at the current imaging resolution (~1 mm^3^), MIX will enable the neuroimaging community to investigate the microstructure of the brain white matter in ways which are currently not possible.

We have shown that MIX is a superior alternative to methods that rely on grid search or other common optimization methods. MIX exploits the structure of the problem and is more robust than all other methods. Furthermore, it does not assume single fiber orientations and can directly estimate microstructure parameters in brain areas with complex geometry. Nonetheless, in brain areas with one primary fiber pathway, we have also shown that MIX outperforms other techniques. MIX is broadly applicable to many already available dMRI datasets, such as the Human Connectome Project (HCP). Using HCP data, we have demonstrated its ability to fit a generalized version of the NODDI model (called NODDIx) to estimate axonal orientation dispersions, volume fractions and orientations in up to two pathways. Finally, MIX has a simple and self-contained implementation in MATLAB which has not yet been optimized for computational efficiency, but has been designed for ease in dealing with realistic and generic biophysical models.

We note that recent works have focused on models describing the diffusion signal directly, which can subsequently be used to identify microstructural features such as those detailed in our paper. These techniques include Q-ball imaging^32^,^33^, DSI^34^ (Diffusion Spectrum MR Imaging), DKI^35^ (Diffusion Kurtosis Imaging), SHORE^36^ (Simple Harmonic Oscillator Based Reconstruction and Estimation), MAP^37^,^38^ (Mean Average Propagator) and MAPL^39^ (Laplacian- regularized MAP). Although this paper focuses on the direct estimation of microstructural features from biophysical models, we would like to emphasize that MIX could be applied to the diffusion signal models which rely on weighted sums of exponential functions.

We hope that our proposed method might provide a new, more flexible computational framework to revisit and expand studies on biophysical models evaluation and classification^17^,^18^, to improve current models^40^,^41^, and to develop future contests such as the *White Matter Modeling Challenge*^42^ (http://cmic.cs.ucl.ac.uk/wmmchallenge/).

## Methods

### Problem Formulation

Define 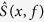 as the model predicted normalized dMRI signal from ‘*n*’ different tissue compartments i.e.


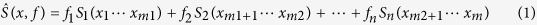


where 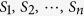 are selected biophysical models for intra-axonal, extra-axonal, cerebrospinal fluid (CSF) and glial cells compartments^6^,^7^,^8^,^9^,^10^,^11^,^12^,^13^,^14^ etc. These models depend upon ‘*m*’ different parameters given in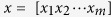, while 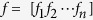 is the vector containing volume fractions of the ‘*n*’ tissue compartments. Objective function for the model parameter estimation from dMRI data, with implicit assumption of offset Gaussian noise as used in the literature^17^,^18^,^21^, is as follows:


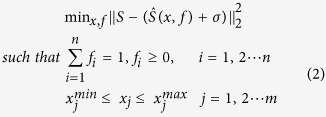


where ‘*S*’ represents the normalized dMRI measurements and ‘σ’ is the standard deviation of noise (assumed to be a constant, calculated a priori, and without loss of generality can be added to *S*). As defined in equation (1), 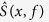
represents the estimated signal from multi-compartment tissue model, while *f*_*i*_s are volume fractions of ‘*n*’ compartments. 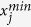 and 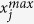 represent lower and upper bounds for unknown deterministic variable vector ‘*x*’ respectively. Diffusion weighted MRI data inherently has Rician noise^3^. However, with an assumption of offset Gaussian noise model, the objective function, as given in equation (2), becomes simple and more stable numerically than Rician log-likelihood function^17^,^18^,^21^.

### Solution Framework

MIX can be divided into four main steps. First separate linear parameters (*f*) from the non-linear parameters (*x*) by projecting variables. The parameters ‘*x*’ estimation problem is a non-linear one and can be solved by stochastic search procedures, here we use Genetic Algorithms (GA). Once estimates of ‘*x*’ are obtained, searching for ‘*f  *’ is a linear least-squares estimation problem. Finally, using estimates from step 2 and 3, as starting point, we perform search for both ‘*x*’ and ‘*f  *’ using the Trust Region Method. Below is the detailed description of each step:

- Step 1 - Variable Separation: The objective function described in equation (2) has a separable structure. Which can be exploited to separate the variables by variable separation method^26^. We can re-write our objective function in equation (2) in the following form:
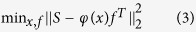
where 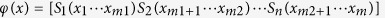.Also it can be noted that
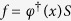
where 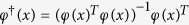 is the Moore-Penrose inverse of *φ(x*). After the projection, the objective function takes the following form:
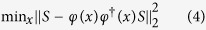
Equation (4) is called the variable projection functional. Assuming that *ϕ(x*) has a locally constant rank, it has been proven^27^ that the global minimum of equation (4) remains the same as the global minimum of equation (3).Rank Constancy of *φ (x*): The matrix *φ(x*) has much larger number of rows (measurements) than the number of columns (compartments). Furthermore, the measurements are always noisy. Thus generally, it is safe to assume that *φ(x*) will always have full column rank.
- Step 2 - Stochastic search for non-linear parameters ‘*x*’: The objective function given in equation (4) is non-convex, particularly of non-linear least-square form. Any gradient based method employed to estimate the parameters will have critical dependence on a good starting point, which is unknown. Alternative approach can be regular grid search, which is time consuming and adds computational burden. This particular type of problem therefore points towards considering stochastic search methods like GA. In case of time series analysis, it has been shown^28^ that GA can be used efficiently for sum of exponentials functions. GA parameters can be varied for each selected biophysical model and time complexity may change with each choice. However, we found that these do not have substantial effect on convergence both in terms of accuracy and time complexity. For all the experiments and results reported in this study, we keep GA parameters in the following range (1) GA method: Elitism based (2) Population size: 24 to 48 (3) Stopping criteria: fixed to 90 iterations (generations) (4) Generation gap: fixed to 0.7.
- Step 3 - Constrained search for linear parameters ‘*f  *’: After estimating the parameters ‘*x*’, estimation of linear parameters ‘*f  *’ is a constrained linear least-squares estimation problem as shown below:
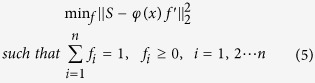
We used CVX (http://cvxr.com/cvx) for solving the optimization problem in equation (5).
- Step 4 - Non Linear Least Squares Estimation using Trust Region Method: Step 2 and step 3 give a reliable initial guess of both ‘*x*’ and ‘*f  *’ to solve equation (2) by applying Trust Region method. Particularly, MATLAB’s ‘lsqcurvefit’ was used to solve the constrained NLLS estimation problem in equation (2). This guarantees that stopping point of the algorithm is at least a stationary point. Also, it reduces the number of iterations (generations) and population size used in the GA (step 2). For example, in time series analysis to have precise results, GA requires population size of 250 with 150 iterations^28^. With the type of functions describing white matter multi-compartment biophysical models, such a stochastic search will become computationally prohibitive.

#### Code availability

MATLAB code for MIX is available online http://www.cmrr.umn.edu/downloads/mix/. ActiveAx, NODDI, ZCDx and NODDIx experiments presented in this article are readily reproducible using this code and the instructions provided therein.

## Additional Information

**How to cite this article**: Farooq, H. *et al*. Microstructure Imaging of Crossing (MIX) White Matter Fibers from diffusion MRI. *Sci. Rep.* **6**, 38927; doi: 10.1038/srep38927 (2016).

**Publisher's note:** Springer Nature remains neutral with regard to jurisdictional claims in published maps and institutional affiliations.

## Supplementary Material

Supplementary Information
