# Supplementary material for: Microstructure Imaging of Crossing (MIX) White Matter Fibers from diffusion MRI
Source: Sci Rep. 2016 Dec 16;6:38927. doi: 10.1038/srep38927 (PMC5159854; doi:10.1038/srep38927)
Supplement: Supplementary Information [file srep38927-s1.pdf]

Microstructure Imaging of Crossing (MIX) White Matter Fibers from diffusion MRI

Hamza Farooq[1*], Junqian Xu[2], Jung Who Nam[3], Daniel F. Keefe[3], Essa Yacoub[4], Tryphon Georgiou[5] & Christophe Lenglet[4]

[1] *Department of Electrical and Computer Engineering, University of Minnesota, Minneapolis, MN, USA.*
[2] *Department of Radiology, Icahn School of Medicine at Mount Sinai, New York, NY, USA.*
[3] *Department of Computer Science and Engineering, University of Minnesota, Minneapolis, MN, USA.*
[4] *Center for Magnetic Resonance Research, Department of Radiology, University of Minnesota, Minneapolis, MN, USA.*
[5] *Department of Mechanical and Aerospace Engineering, University of California, Irvine, CA, USA.*

SUPPLEMENTARY NOTES

Supplementary Note 1: Tissue Compartment Model Functions

Here we present compartment model functions describing the dMRI signal attenuation due to diffusion in a certain tissue geometry. We limit the description to the functions used in our work, please refer to [1] for a more comprehensive list of available compartment model functions. We describe the parameters of the model functions and the Pulse Gradient Spin Echo sequence in paragraph 1 and 2 respectively, followed by the model functions in paragraph 3.

1. **Parameters of the Compartment Model Functions**
   a. **Fiber orientation vector:** Fiber (axon) orientation vector '*n*' is given by Eq. (1.1), where θ, φ and α are angles (in radians) as shown in Supplementary Fig. S1.1.

$$n = [\cos\varphi \sin\theta \,;\; \sin\varphi \cos\theta \;;\; \cos\theta] \qquad (1.1)$$

   b. $\boldsymbol{d}_{\parallel}$ $(\mu m^2.sec^{-1})$ = Diffusivity in direction parallel to '*n*' (intrinsic free diffusivity).
   c. $\boldsymbol{d}_{\perp 1}$ $(\mu m^2.sec^{-1})$ = Diffusivity in direction perpendicular to '*n*'.
   d. $\boldsymbol{d}_{\perp 2}$ $(\mu m^2.sec^{-1})$ = Diffusivity in direction perpendicular to '*n*' and $d_{\perp 1}$.
   e. $\boldsymbol{d}_{iso}$ $(\mu m^2.sec^{-1})$ = Isotropic diffusivity, fixed to$2.0e^3$ $(\mu m^2.sec^{-1})$ for ex-vivo and $3.0e^3$ $(\mu m^2.sec^{-1})$ for in-vivo.
   f. $\boldsymbol{R}$ (μm) = Axon radius index [2].
   g. $\boldsymbol{v}_{ic}$ = Intra-cellular volume fraction [3].
   h. $\boldsymbol{v}_{iso}$ = Isotropic volume fraction [3].
   i. $\boldsymbol{OD}$ = Orientation dispersion index [3].

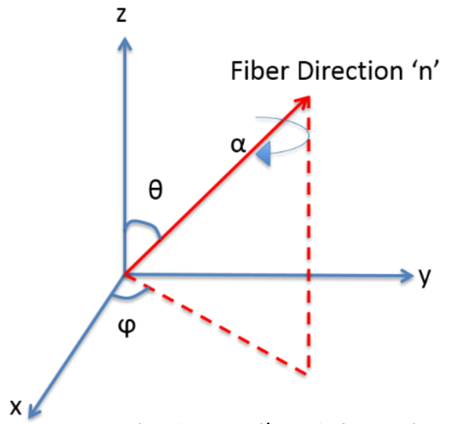

Supplementary Fig. S1.1 - Fiber Orientation

2. **Parameters of the Pulse Gradient Spin Echo (PGSE) Sequence:** PGSE parameters [4] are given below, Supplementary Fig. S1.2 shows the parameters describing the pulse sequence.
   a. $\boldsymbol{G}\ (Tesla.\mu m^{-1})$ = Gradient strength.
   b. $\boldsymbol{g} = [\boldsymbol{g_x}\ ;\ \boldsymbol{g_y}\ ;\ \boldsymbol{g_z}]$ Gradient direction (unit) vector.
   c. $\boldsymbol{\delta}\ (sec)$ = Pulse width.
   d. $\Delta\ (sec)$ = Diffusion time.
   e. $\boldsymbol{\gamma}\ (rad\ (\mathrm{sec}.Tesla)^{-1})$ = Gyro-magnetic ratio.
   f. $\boldsymbol{b}\ (sec.\mu m^{-2})$ = Diffusion weighting factor, also called *b*-value.

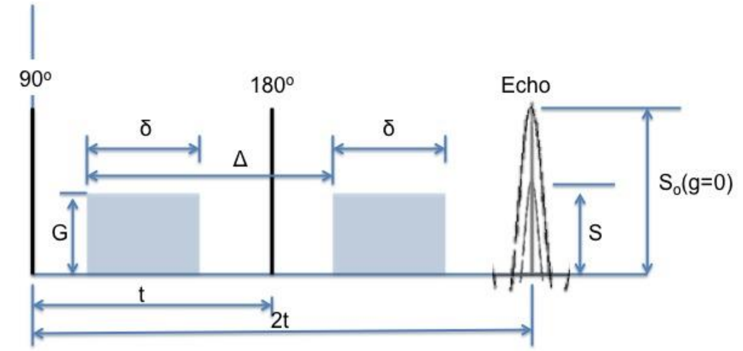

Supplementary Fig. S1.2 - Pulse Gradient Spin Echo Sequence (PGSE)

3. **Biophysical Functions for Compartment Models.**

   a. **Zeppelin model function**

$$S_{zeppelin} = e^{-b(d_{\parallel}-d_{\perp})(g.n)^2+d_{\perp}} \quad (1.2)$$

   b. **Tensor model function**

$$S_{tensor} = e^{-bg'Dg} \quad (1.3)$$

Where $D \rightarrow S^3_{++}$ (symmetric positive definite 3 x 3 matrix)

   c. **Cylinder model function**

$$S_{cylinder} = e^{-S_{cyl\parallel}}.e^{-S_{cyl\perp}} \quad (1.4)$$

$$S_{cyl\parallel} = \Delta - (\delta/3)(\gamma\delta G)^2 d_{\parallel} \quad (1.4.1)$$

$$S_{cyl\perp} = 2\gamma^2(G^2 - (G.n)^2)\sum_{i=1}^{\infty}\frac{2d_{\parallel}\beta_i^2\delta - 2 + 2e^{-d_{\parallel}\beta_i^2\Delta} - e^{-d_{\parallel}\beta_i^2(\Delta-\delta)} - e^{-d_{\parallel}\beta_i^2(\Delta+\delta)} + 2e^{-d_{\parallel}\beta_i^2\delta}}{d_{\parallel}^2\beta_i^6(\beta_i^2R^2 - 1)} \quad (1.4.2)$$

Where β_i is the ith root of the equation, $J'(\beta_i R) = 0$, where J' is the derivative of Bessel function of the first kind, order one.

   d. **Ball model function**

$$S_{ball} = e^{-bd_{iso}} \quad (1.5)$$

   e. **Stick model function**

$$S_{stick} = e^{-bd_{\parallel}(nG)^2} \quad (1.6)$$

f. **Dot model function**

$$S_{dot} = e^{-b_0=0} = 1 \tag{1.7}$$

References:

[1]. Panagiotaki, E. et al. *Neuroimage* **59**, 2241-2254 (2012).
[2]. Alexander, D.C. et al. *Neuroimage* **52**, 1374-1389 (2010).
[3]. Zhang et al. *Neuroimage* **61**, 1000-1016 (2012).
[4]. J.E. Tanner and E. O. Stejskal. *J. Chem. Phys*. **49**, 1768 (1968).

Supplementary Note 2: Synthetic Data Experiments

We performed synthetic data experiments using four different tissue models. Settings for all four experiments are given below:

1. **Experiment 1 – ActiveAx model fitting.** ActiveAx is a four compartment model i.e, Zeppelin-Cylinder-Ball-Dot [1,2]. The model function showing only the parameters to be estimated (unknown parameters) is given below:
   a. **Model function**: The estimated dMRI normalized signal $\hat{S}_{ActiveAx}$ is assumed to be coming from the following four compartments:
   $$\hat{S}_{ActiveAx} = f_1 S_{cylinder}(R, \theta, \varphi) + f_2 S_{zeppelin}(d_{\perp}, \theta, \varphi) + f_3 S_{ball} + f_4 S_{dot} \quad (2.1)$$
   Where $f_i s$ are the volume fractions of each compartment such that $\sum_{i=1}^{4} f_i = 1$. Remaining parameters have been defined in **Supplementary Note 1**.
   b. **Parameters to be estimated**: Eight $(f_1, f_2, f_3, f_4, R, d_{\perp}, \theta, \varphi)$.
   c. **Generating synthetic data**: Synthetic data was generated using CAMINO "datasynth" command for 100 substrates. True values (ground truth values) of the parameters were randomly and uniformly generated over the parameter space (as given below), using MATLAB:
   $$f_i \in [0.01, 1] \text{ such that } \sum_{i=1}^{4} f_i = 1$$
   $$R \in [0.1, 10]\ (\mu m) \quad , \quad \theta, \varphi \in [0.01, \pi]\ (rad)$$
   $$d_{\perp} = (1 - f_1)\, d_{\parallel}\ (\mu m^2.sec^{-1}) \text{ as in [3]}$$
   $d_{\parallel} = 0.6e^3\ (\mu m^2.sec^{-1})$ and $d_{iso} = 2.0e^3\ (\mu m^2.sec^{-1})$ were fixed for ex-vivo as in [1,2].
   d. **Noise**: Rician noise was added to signal for each substrate with SNR=1000, 20 and 8 with ten different realizations for each SNR value, i.e., we have 3000 different noise realizations.
   e. **Protocol**: An optimized protocol for ActiveAx given in the original study [2] was used for synthetic data generation and subsequently for parameter estimation. The protocol has four shells and a total of 372 measurements (including b_0 non-diffusion weighted measurements).
   f. **Results**: Given as **Supplementary Figure 1**.

2. **Experiment 2 – NODDI model fitting**
   a. **Model function**: As introduced in [4], estimated dMRI signal $\hat{S}_{Noddi}$ comprises of the normalized signals from the following three compartments:
   $$\hat{S}_{Noddi} = (1 - v_{iso})(v_{ic} S_{ic}(OD, \theta, \varphi) + (1 - v_{ic}) S_{ec}(d_{\perp}, \theta, \varphi) + v_{iso} S_{dot} \quad (2.2)$$
   Where S_{ic} and S_{ec} are the normalized signals from intra-cellular and extra-cellular compartments respectively. Details for the remaining parameters are given in **Supplementary Note 1**.
   b. **Parameters to be estimated**: Six $(v_{ic}, v_{iso}, OD, d_{\perp}, \theta, \varphi)$.
   c. **Generating synthetic data**: Synthetic data was generated using 'WatsonSHStickTortIsoV-B0' model i.e. using NODDI MATLAB model functions 'SynthMeasWatsonSHCylNeuman-PGSE' as intra-cellular model, 'SthMeasWatsonHinderedDiffusion-PGSE' as extra-cellular model and S_{dot} as isotropic compartment model. Ground truth values for the six parameters were generated randomly and uniformly over the following parameter space:
   $$v_{ic},\ v_{iso} \in [0.01, 0.9] \quad , \quad OD \in [0.1, 1] \quad , \quad \theta, \varphi \in [0.01, \pi]\ (rad)$$
   $d_{\parallel} = 1.7e^3\ (\mu m^2.sec^{-1})$ and $d_{iso} = 3.0e^3\ (\mu m^2.sec^{-1})$ were fixed for in-vivo as in [3,4].

d. **Noise**: Rician noise was added to each substrate with SNR=1000, 20 and 8. Ten different noise realizations for each SNR value were added to the substrates giving 3000 different noise realizations.
e. **Protocol**: We used the optimized protocol for NODDI given in original study [4] for synthetic data generation and parameter estimation. The protocol has two b-values and a total of 81 measurements (including b_0 measurements).
f. **Results**: Given as **Supplementary Figure 2**.

3. <u>**Experiment 3 – Tensor-Stick-Dot (TSD) model fitting.**</u>
   a. **Model function:** The estimated dMRI signal $\hat{S}_{TSD}$ function is:

$$\hat{S}_{TSD} = f_1 S_{stick}(d_{\parallel}, \theta, \varphi) + f_2 S_{tensor}(d_{\parallel}, d_{\perp 1}, d_{\perp 2}, \theta, \varphi, \alpha) + f_3 S_{dot} \quad (2.3)$$

Unlike experiments 1 and 2, here we do not fix values for $d_{\parallel}$, $d_{\perp 1}$ and $d_{\perp 2}$. As explained earlier, $f_i s$ are the volume fractions of each compartment such that $\sum_{i=1}^{3} f_i = 1$. All other parameters have been defined in **Supplementary Note 1**.

   b. **Parameters to be estimated**: Nine $(f_1, f_2, f_3, d_{\parallel}, d_{\perp 1}, d_{\perp 2}, \theta, \varphi, \alpha)$.
   c. **Generating synthetic data**: Similar to experiment 1, synthetic data was generated using CAMINO "datasynth" command for 100 substrates. Using MATLAB, ground truth values of the parameters were randomly and uniformly generated over the parameter space, which is given below:

$$f_i \in [0.01, 1] \text{ such that } \sum_{i=1}^{3} f_i = 1 \quad , \qquad \theta, \varphi, \alpha \in [0.01, \pi]\ (rad)$$

$$d_{\parallel}, d_{\perp 1}, d_{\perp 2} \in [0.55e^3\ , 1.8e^3]\ (\mu m^2.sec^{-1}) \quad \text{while} \quad d_{\parallel} > d_{\perp 1} > d_{\perp 2}$$

   d. **Noise**: Ten different noise realizations (at SNR=1000) were generated and added to each substrate to have 1000 different noise realizations in total.
   e. **Protocol**: Since no optimized protocol is available for the model, we used the ActiveAx protocol [2] (as in experiment 1).
   f. **Results**: Given as **Supplementary Figure 3**.

4. <u>**Experiment 4 – Zeppelin-Cylinder-Dot in three orientations (ZCDx) model fitting.**</u>
   a. **Model function:** In order to demonstrate the ability of our method to recover axonal microstructure information in complex white matter configurations, we hypothesize three fiber orientations in a voxel. Specifically, we assume that a zeppelin and a cylinder representing extra-axonal and intra-axonal signal respectively, in each of the three orientations. This can be seen as an extension of ActiveAx [2] model for three different fiber orientations in a voxel. The estimated dMRI signal $\hat{S}$ is assumed to be composed of normalized signals from the following seven compartments:

$$\hat{S}_{ZCDx} = \underbrace{\overbrace{f_{11} S_{cylinder1}(R_1, \theta_1, \emptyset_1)}^{Intra-axonal1} + \overbrace{f_{12} S_{zeppelin1}(d_{\perp 1}, \theta_1, \emptyset_1)}^{Extra-axonal1}}_{Primary\ Orientation} + \underbrace{\overbrace{f_{21} S_{cylinder2}(R_2, \theta_2, \emptyset_2)}^{Intra-axonal2} + \overbrace{f_{22} S_{zeppelin2}(d_{\perp 2}, \theta_2, \emptyset_2)}^{Extra-axonal2}}_{Secondary\ Orientation}$$
$$+ \underbrace{\overbrace{f_{31} S_{cylinder3}(R_3, \theta_3, \emptyset_3)}^{Intra-axonal3} + \overbrace{f_{32} S_{zeppelin3}(d_{\perp 3}, \theta_3, \emptyset_3)}^{Extra-axonal3}}_{Tertiary\ Orientation} + \underbrace{f_3 S_{dot}}_{CSF\ Compartmet\ (Dot)} \quad (2.4)$$

   b. **Parameters to be estimated**: Nineteen, give as under:

$$(f_{11}, f_{12}, f_{21}, f_{22}, f_{31}, f_{32}, f_3, R_1, d_{\perp 1}, \theta_1, \emptyset_1, R_2, d_{\perp 2}, \theta_2, \emptyset_2, R_3, d_{\perp 3}, \theta_3, \emptyset_3)$$

   c. **Generating synthetic data**: Synthetic data was generated using CAMINO "datasynth" command for 100 substrates. True values (ground truth values) of the parameters were randomly and uniformly generated over the parameter space (as given below), using MATLAB:

$$f_{ik} \in [0.01, 1] \text{ such that } \sum_{i=1}^{3} \sum_{k=1}^{4} f_{ik} + f_3 = 1$$

$$R_k \in [0.1, 10]\ (\mu m) \quad , \quad \theta_k, \varphi_k \in [0.01, \pi]\ (rad)$$

$d_{\parallel k} = 1.7e^3\ (\mu m^2.sec^{-1})$ and $d_{\perp k} = (1 - f_{1k})\ d_{\parallel k}\ (\mu m^2.sec^{-1})$ were fixed for in-vivo as in [1-3]. Where $k = 1,2,3$ represent number of fiber orientations inside a voxel.

d. **Noise**: Similar to all the experiments above, Rician noise was added to each substrate with SNR=1000 and SNR=20 i.e., we have 2000 different noise realizations.

e. **Protocol**: Protocol used for this experiment was made available for the White Matter Modeling Challenge (http://cmic.cs.ucl.ac.uk/wmmchallenge/) for predicting dMRI data. The protocol has 36 shells and a total of 3612 measurements (including b_0 measurements).

f. **Results**: Given as **Supplementary Figure 4-5**.

References:

[1]. Panagiotaki, E. et al. *Neuroimage* **59**, 2241-2254 (2012).

[2]. Alexander, D.C. et al. *Neuroimage* **52**, 1374-1389 (2010).

[3]. Daducci, A. et al. *Neuroimage* **105**, 32-44 (2015).

[4]. Zhang et al. *Neuroimage* **61**, 1000-1016 (2012).

Supplementary Note 3: Time Complexity Comparison on Synthetic Data

The estimation time depends upon a number of factors like the underlying model, number of dMRI measurements available, and in the case of CAMINO also on noise level. Following is the time complexity comparison for synthetic data experiments using ActiveAx, NODDI and Tensor-Stick-Dot models. (Note: for all methods we include time for data loading and processing/pre-processing). Times were obtained without any parallel processing unless indicated (Core i7 with 12 GB RAM).

1. **ActiveAx Model**: Fitting time for 100 substrates of synthetic data, is as follows:
   a. **CAMINO**: 40 - 45 minutes.
   b. **MIX**: 1000 seconds approximately.
   c. **AMICO**: 250 seconds approximately.

2. **NODDI Model**: MATLAB 2012b was used for the experiment as later MATLAB versions lead to longer time with NODDI implementation. For details please see discussion at NODDI Google group: (https://groups.google.com/forum/#!searchin/noddi/Avoid$20MATLAB$202013b$20onwards/noddi/oPkZamvpZ3Q/FRHpN0-hFCgJ). Estimation time for 100 substrates for each algorithm is given below:
   a. **MIX**: 592.61 seconds without parallel processing and 63.59 seconds with parallel processing (parallel pool of 12 workers in MATLAB).
   b. **NODDI**: 191.8 seconds without parallel processing and 31.93 seconds with parallel processing (parallel pool of 12 workers in MATLAB).
   c. **AMICO**: AMICO took approximately 6 seconds for data fitting. Data pre-processing like pre-computing rotation matrices (94.7 seconds, once and for all), generating kernels for protocol (393.6 seconds) and re-sampling rotated kernels (90.6 seconds) are additional. As reported in [1], AMICO is the fastest and its estimation time does not scale with data, which is not the case for all other algorithms.
3. **Tensor-Stick-Dot Model**:
   a. **CAMINO**: approximately 11 mins for single run LM and approximately 50 mins for MULTIRUNLM (10 runs) and about 5 hours for 100 runs (for results shown in **Supplementary Fig. 3**).
   b. **MIX**: 113 seconds approximately (for the results shown in **Supplementary Fig. 3**).

References:

[1]. Daducci, A. et al. *Neuroimage* **105**, 32-44 (2015).

Supplementary Note 4: Intracellular volume fraction (v_{ic}) estimation for NODDI model with MIX

NODDI Model function: As described in the original study [1], NODDI model function is given as Eq. 4.1. Details on the function have already been provided in Supplementary Note 2. For estimation using MIX, we re-write Eq. 4.1 as 4.2 to separate variables. Also we introduce constraint as in Eq. 4.3, which has not been implemented previously. If we get large f_3 (or v_{iso}), f_1 and f_2 consequently will be very small. This can result in large v_{ic} in turn, which is obvious due to Eq. 4.4 or Eq. 4.5 (Supplementary Figures S4.1 - S4.3). So a large value of v_{ic} is relative to extra-cellular volume fraction only and not to v_{iso}.

$$(1 - v_{iso})(v_{ic}A_{ic} + (1 - v_{ic})A_{ec}) + v_{iso}A_{iso} \qquad (4.1)$$

$$\underbrace{(v_{ic} - v_{ic}v_{iso})}_{f_1}A_{ic} + \underbrace{(1 - v_{ic} - v_{iso} + v_{ic}v_{iso})}_{f_2}A_{ec} + \underbrace{v_{iso}}_{f_3}A_{iso} \qquad (4.2)$$

$$\text{such that} \quad f_1 + f_2 + f_3 = 1 \qquad (4.3)$$

$$v_{ic} = \frac{f_1}{1 - v_{iso}} \qquad (4.4)$$

or

$$v_{ic} = 1 - \left(\frac{f_2}{1 - v_{iso}}\right) \qquad (4.5)$$

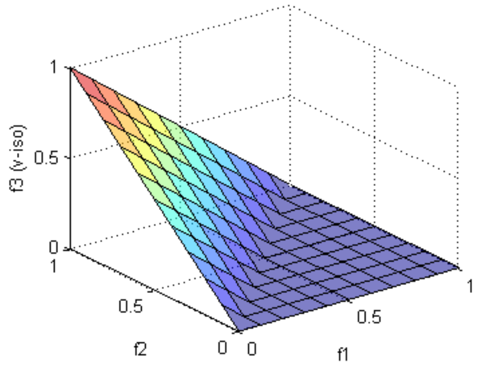

Supplementary Fig. S4.1 showing Eq. 5.3 plot

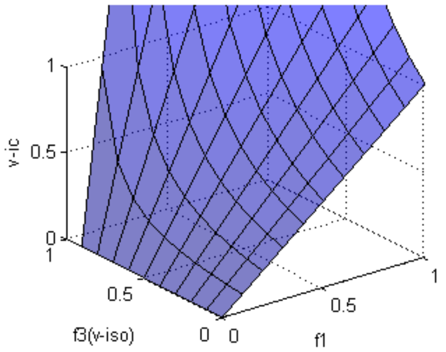

Supplementary Fig. S4.2 - v_{ic} plot for f_1 and f_3

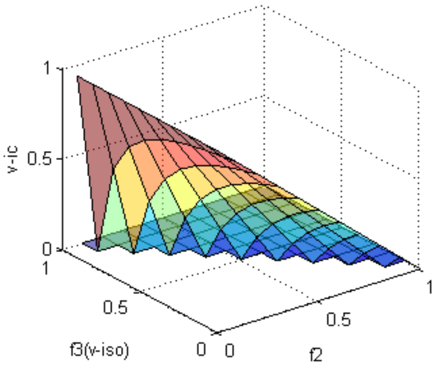

Supplementary Fig. S4.3 - v_{ic} plot for f_2 and f_3

References:

[1]. Zhang et al. *Neuroimage* **61**, 1000-1016 (2012).

Supplementary Note 5: Zeppelin-Cylinder-Cylinder-Dot (ZCCD) model fitting to in-vivo data

1. **Model function:** We assume that a zeppelin represents the extra-axonal compartment, and two cylinders represent intra-axonal compartments. All cylinders and zeppelin can can have arbitray orientations (Supplementary Fig. S5.1). Like ZCDx, this can also be seen as an extension of the ActiveAx [1] model for three different fiber orientations in a voxel. The estimated dMRI signal $\hat{S}$ is assumed to be composed of normalized signals from the following four compartments:

$$\hat{S}_{ZCCD} = \overbrace{\underbrace{f_1 S_{cylinder1}(R_1, \theta_1, \emptyset_1)}_{Primary\ Orientation}}^{Intra-axonal1} + \overbrace{\underbrace{f_2 S_{cylinder2}(R_2, \theta_2, \emptyset_2)}_{Secondary\ Orientation}}^{Intra-axonal2} + \overbrace{\underbrace{f_3 S_{zeppelin}(d_{\perp}, \theta_3, \emptyset_3)}_{Tertiary\ Orientation}}^{Extra-axonal} + \underbrace{f_4 S_{dot}}_{CSF\ Compartmet\ (Dot)} \quad (5.1)$$

2. **Parameters to be estimated**: Thirteen, give as under:

$$(f_1, f_1, f_3, f_4, \theta_1, \emptyset_1, R_1, \theta_2, \emptyset_2, R_2, d_{\perp}, \theta_3, \emptyset_3)$$

3. **Protocol**: For acquiring in-vivo human brain data, protocol used had four b-values, each with 128 directions and 9 additional b=0 measurements. Corresponding parameters are as follows: b_1 = 820 s.mm^{-2} (Δ/δ = 17.6 / 9 ms, |G| = 98.5 mT.m^{-1}), b_2 = 980 s.mm^{-2} (Δ/δ = 55.5 / 5.2 ms, |G| = 97.1 mT.m^{-1}), b_3 = 3010 s.mm^{-2} (Δ/δ = 38.5 / 22.2 ms, |G| = 52.4 mT.m^{-1}) and b_4 = 7600 s.mm^{-2} (Δ/δ =37.8 / 29.3 ms, |G| = 66.6 mT.m^{-1}).

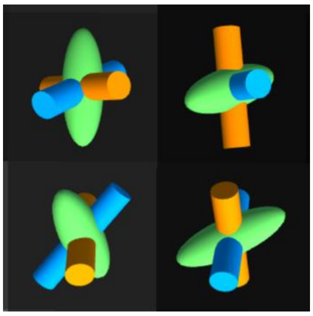

Supplementary Fig. S5.1 – ZCCD model.

References:

[1]. Alexander, D.C. et al. *Neuroimage* **52**, 1374-1389 (2010).

Supplementary Note 6: NODDIx model fitting to Human Connectome Project (HCP) data

1. **Model function**: We extend the NODDI model as presented in [1] for two fiber orientations. dMRI signal $\hat{S}_{Noddix}$ comprises of the normalized signals from the following five compartments:

$$\hat{S}_{Noddix} = \sum_{n=1}^{2} v_{icn} S_{icn}(OD_n, \theta_n, \varphi_n) + v_{ecn} S_{ecn}(d_{\perp n}, \theta_n, \varphi_n) + v_{iso} S_{dot} \quad (6.1)$$

   Where S_{icn} and S_{ecn} are the normalized signals from intra-cellular and extra-cellular compartments in the n-th fiber orientation, respectively. Details for the remaining parameters are given in **Supplementary Note 1**.

2. **Parameters to be estimated**: Thirteen, i.e.,

$$(v_{ic1}, v_{ec1}, v_{ic2}, v_{ec2}, v_{iso}, OD_1, OD_2, \theta_1, \varphi_1, \theta_2, \varphi_2, d_{\perp 1}, d_{\perp 2}).$$

3. **Protocol**: We used HCP protocol for the NODDIx model fitting. The protocol has three b-values and a total of 288 measurements (including b_0 measurements). Details for the protocol can be found in [2].

__**References**__:

[1]. Zhang et al. *Neuroimage* **61**, 1000-1016 (2012).
[2]. Sotiropoulos, S. N. et al. *NeuroImage* **80**, 125–143 (2013).

SUPPLEMENTARY FIGURES

Supplementary Figure 1: ActiveAx parameters estimation using synthetic data

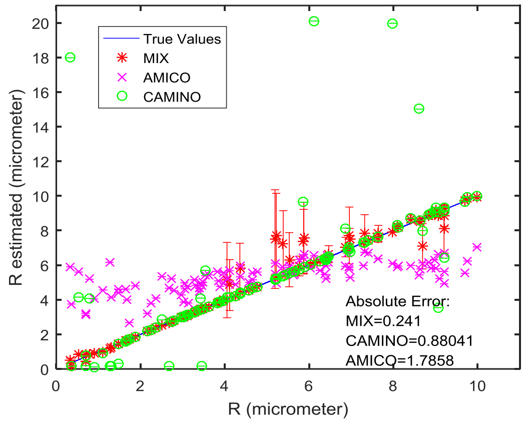

(a) Axon radius index estimates at SNR 1000

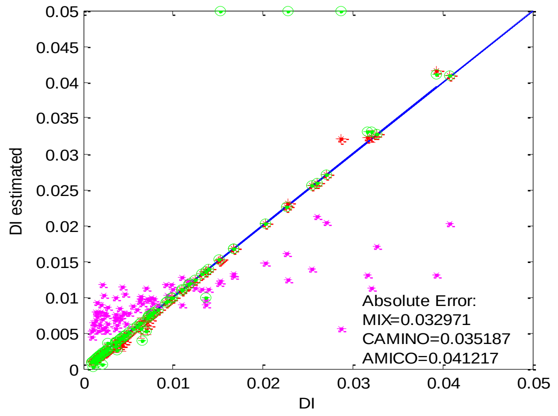

(b) Axon density index estimates at SNR 1000

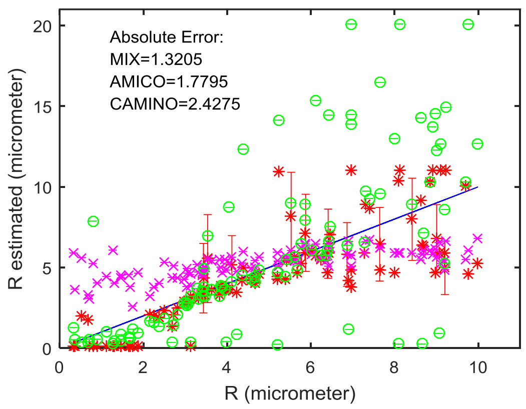

(c) Axon radius index estimates at SNR 20

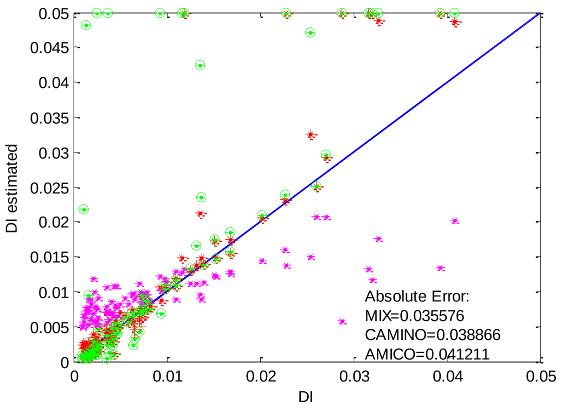

(d) Axon density index estimates at SNR 20

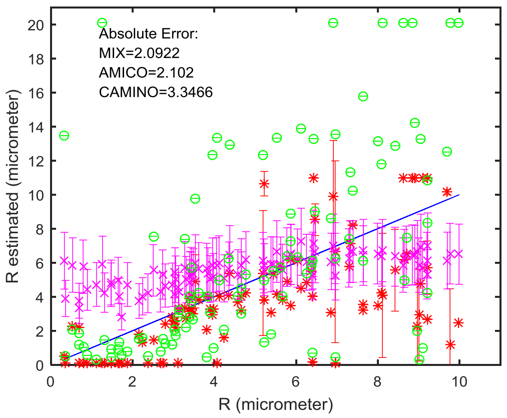

(e) Axon radius index estimates at SNR 8

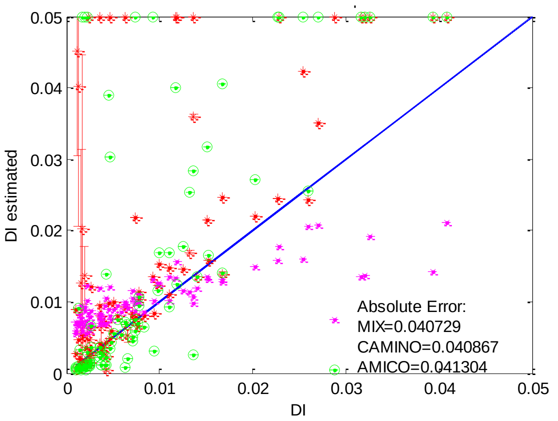

(f) Axon density index estimates at SNR 8

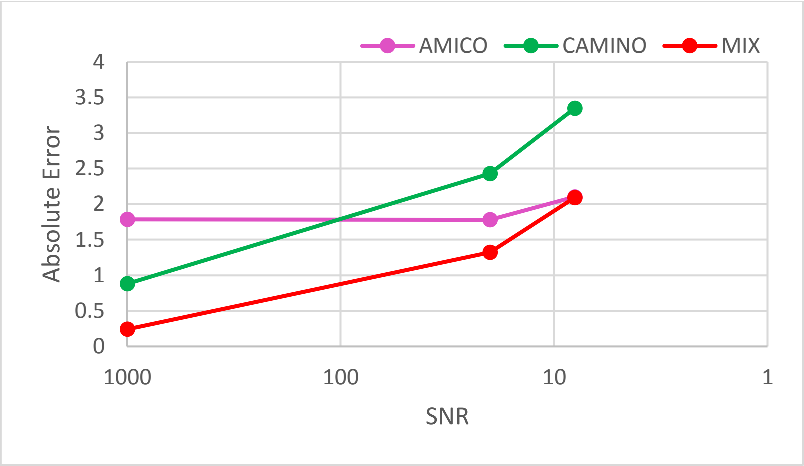

(g) ActiveAx - Axon Radius index estimates - average absolute error

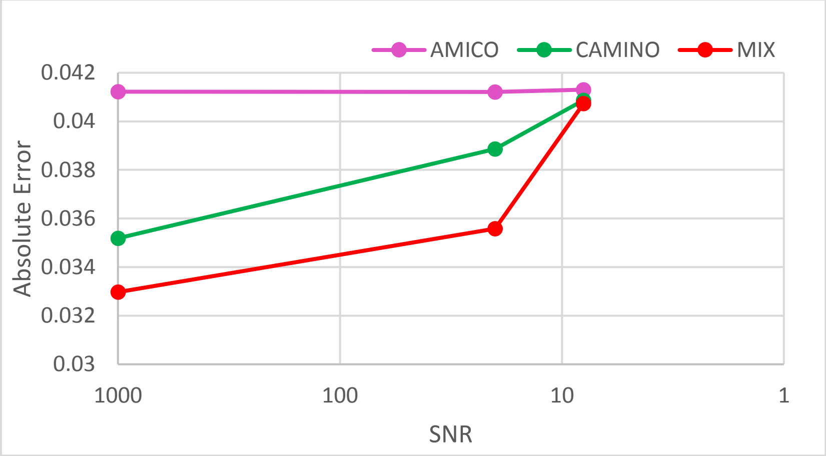

(h) ActiveAx - Axon density index estimates - average absolute error

Supplementary Figure 1. Axon radius index R and axon density index DI [1] estimates comparison at SNR=1000, 20 and 8. The plots are 'errorbars' showing mean and stanfard deviation of the ten estimation results, each with different noise realization for every substrate (i.e., 1000 noise realizations for each noise level). Further, for CAMINO, each estimate is already a mean of 100 samples using MCMC fitting algorithm, as described at CAMINO website (http://camino.cs.ucl.ac.uk/index.php?n=Tutorials.ActiveAx).

All sub-figures show $Absolute\ error = \left|\frac{estimated\ parameter\ values - true\ values}{number\ of\ voxels}\right|$ for each algorithm. Note that DI plots at both SNR levels have been clipped to 0.05 ($axons.\mu m^{-2}$) for all algorithms, which is the range shown in [1-2]. At SNR=1000, R estimates by CAMINO are fairly accurate about 90 percent of the time while AMICO estimates are robust to noise. However, it can be seen that both R and DI have been better estimated by MIX at all noise levels. Supplementary Fig. 1 (g) and (h) give plots of absolute error for each SNR.

References:

[1]. Alexander, D.C. et al. Neuroimage 52, 1374-1389 (2010).
[2]. Daducci, A. et al. Neuroimage 105, 32-44 (2015).

Supplementary Figure 2: NODDI parameters estimation using synthetic data

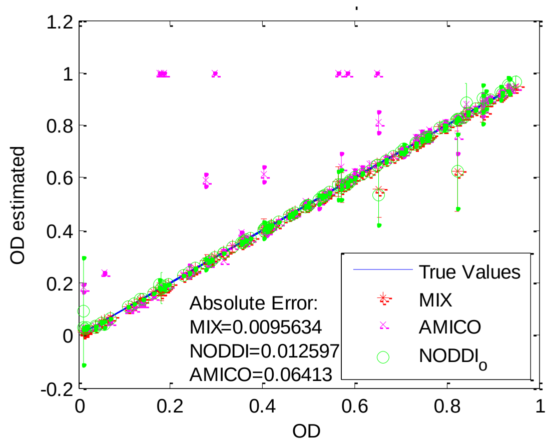

(a) Orientation Disp (OD) estimates at SNR 1000

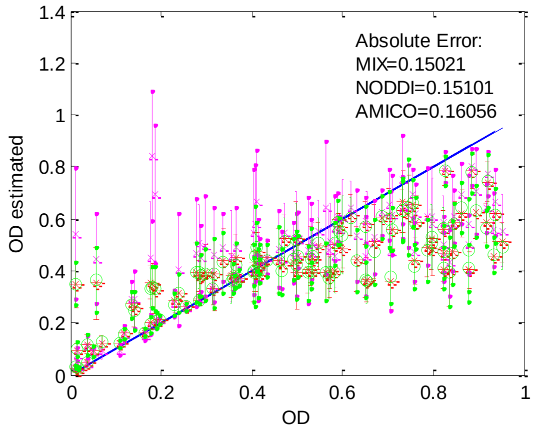

(b) Orientation Disp (OD) estimates at SNR 20

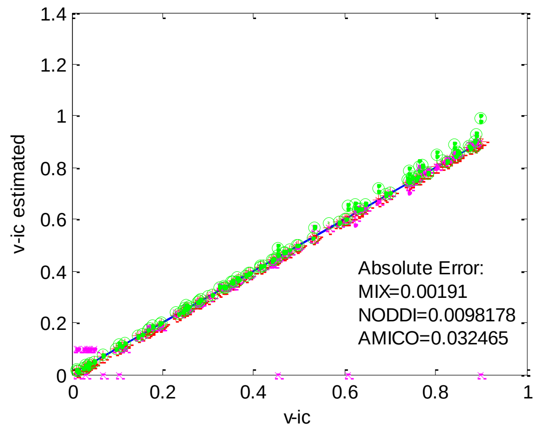

(c) v_{ic} estimates at SNR 1000

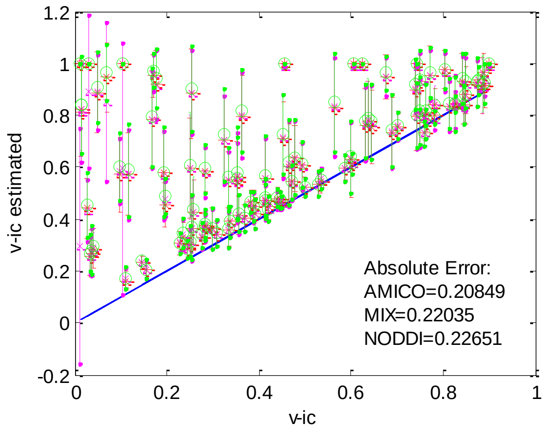

(d) v_{ic} estimates at SNR 20

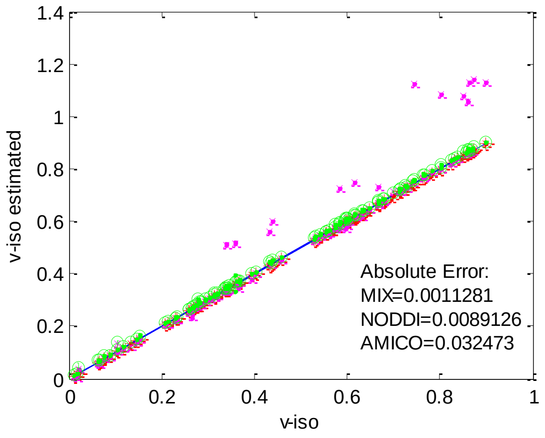

(e) v_{iso} estimates at SNR 1000

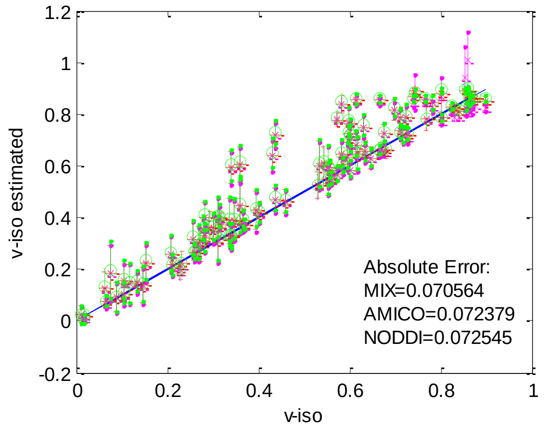

(f) v_{iso} estimates at SNR 20

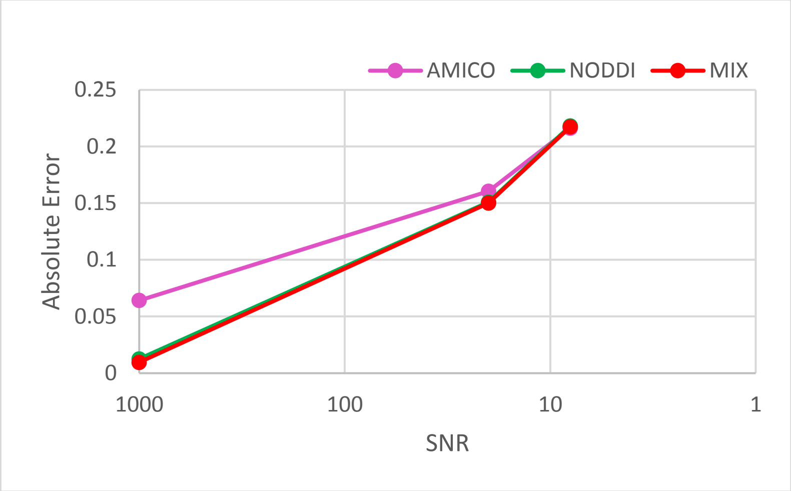

(g) NODDI - Orientation Dispersion (*OD*) - average absolute error

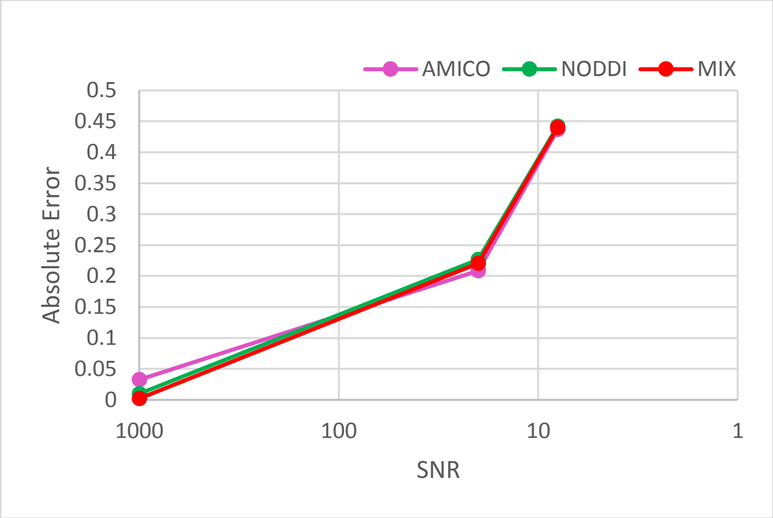

(h) NODDI – Intra-cellular volume fraction (v_{ic}) - average absolute error

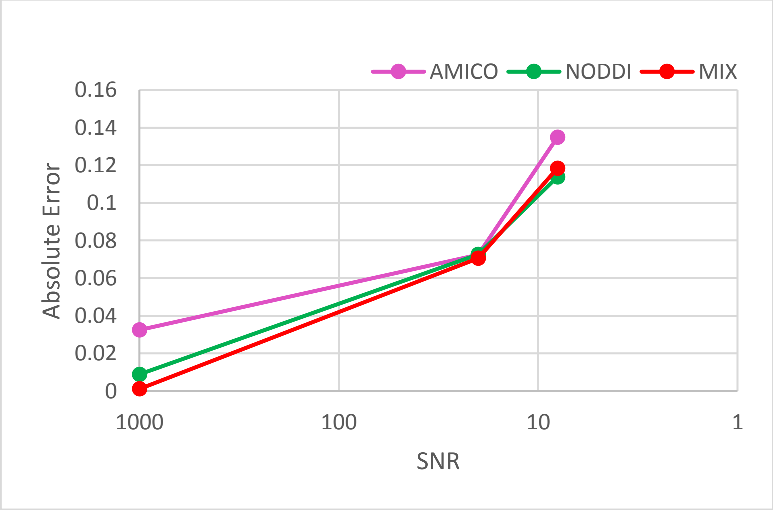

(i) NODDI – Isotropic volume fraction v_{iso} - average absolute error

Supplementary Figure 2. NODDI parameters estimation comparison for the three algorithms. The plots (Supplementary Fig. 2 a – f) are 'errorbars' showing mean and standard deviation of ten

estimation results, each with different noise realization for every substrate (i.e., 1000 noise realizations). Axonal orientation dispersion OD and Intracellular volume fraction v_{ic} estimation results are in-line with the results reported in Fig. 7. of ref [1], i.e., with the increase in noise, higher values of OD are under-estimated while lower values of v_{ic} are over-estimated. Absolute errors of each algorithm have been reported in each sub-figure for comparison. It can be seen that MIX has the least error except for v_{ic} at SNR=20 estimation only. Supplementary Fig. 2 g – i show absolute error plots for SNR=1000, 20 and 8.

References:

[1]. Daducci, A. et al. Neuroimage 105, 32-44 (2015).

Supplementary Figure 3: Tensor-Stick-Dot parameters estimation using synthetic data

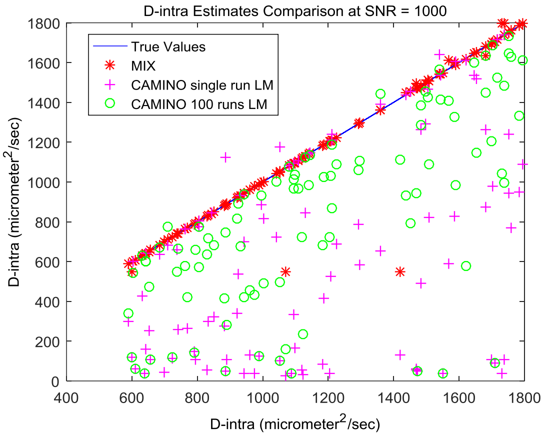

(a) $\boldsymbol{d}_{\parallel}$ (Intrinsic diffusivity) estimates

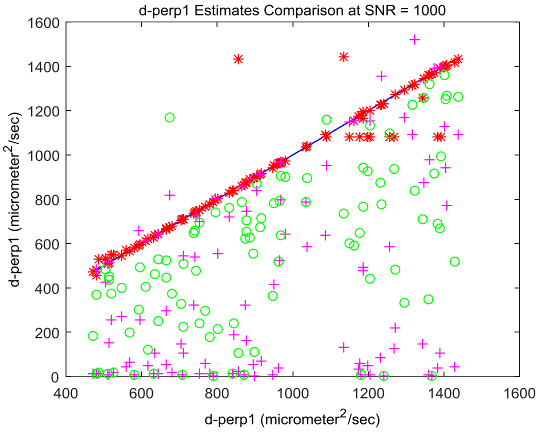

(b) $\boldsymbol{d}_{\perp 1}$ estimates

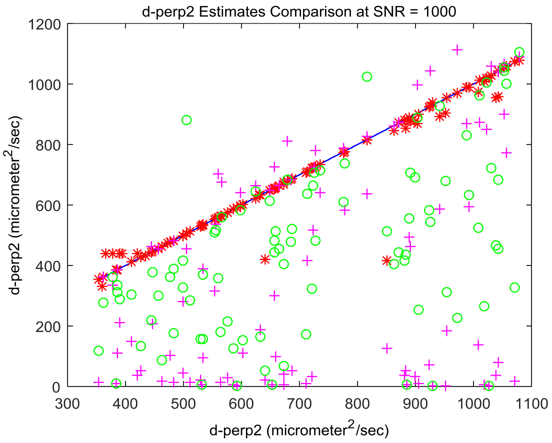

(c) $\boldsymbol{d}_{\perp 2}$ estimates

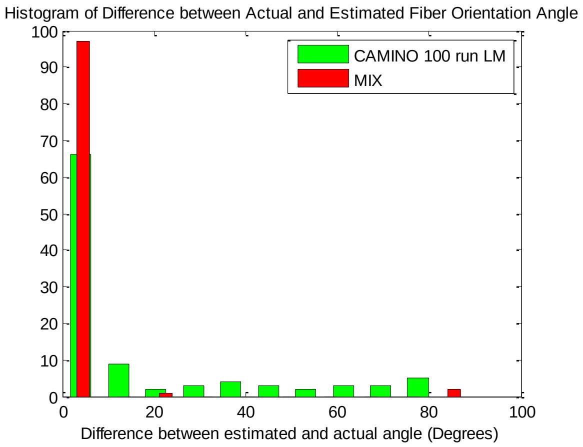

(d) Histogram of difference between angles

Supplementary Figure 3. Tensor-Stick-Dot parameter estimation comparison betweeen MIX and CAMINO at SNR=1000. MIX results shown are for single estimation run. For CAMINO, results are shown for single run Levenberg–Marquardt (LM) and 100 runs LM (using CAMINO's "MULTIRUNLM"). Subfigures (a), (b) and (c) show estimates of $d_{\parallel}$, $d_{\perp 1}$ and $d_{\perp 2}$ ($\mu m^2.sec^{-1}$) respectively while (d) shows fiber orientation estimation comparison (in degrees). It can be seen that all parameters are more accurately estimated in less time (**Supplementary Note 2**) by MIX, as compared to CAMINO.

Supplementary Figure 4: ZCDx parameters estimation using synthetic data (Radius index estimates)

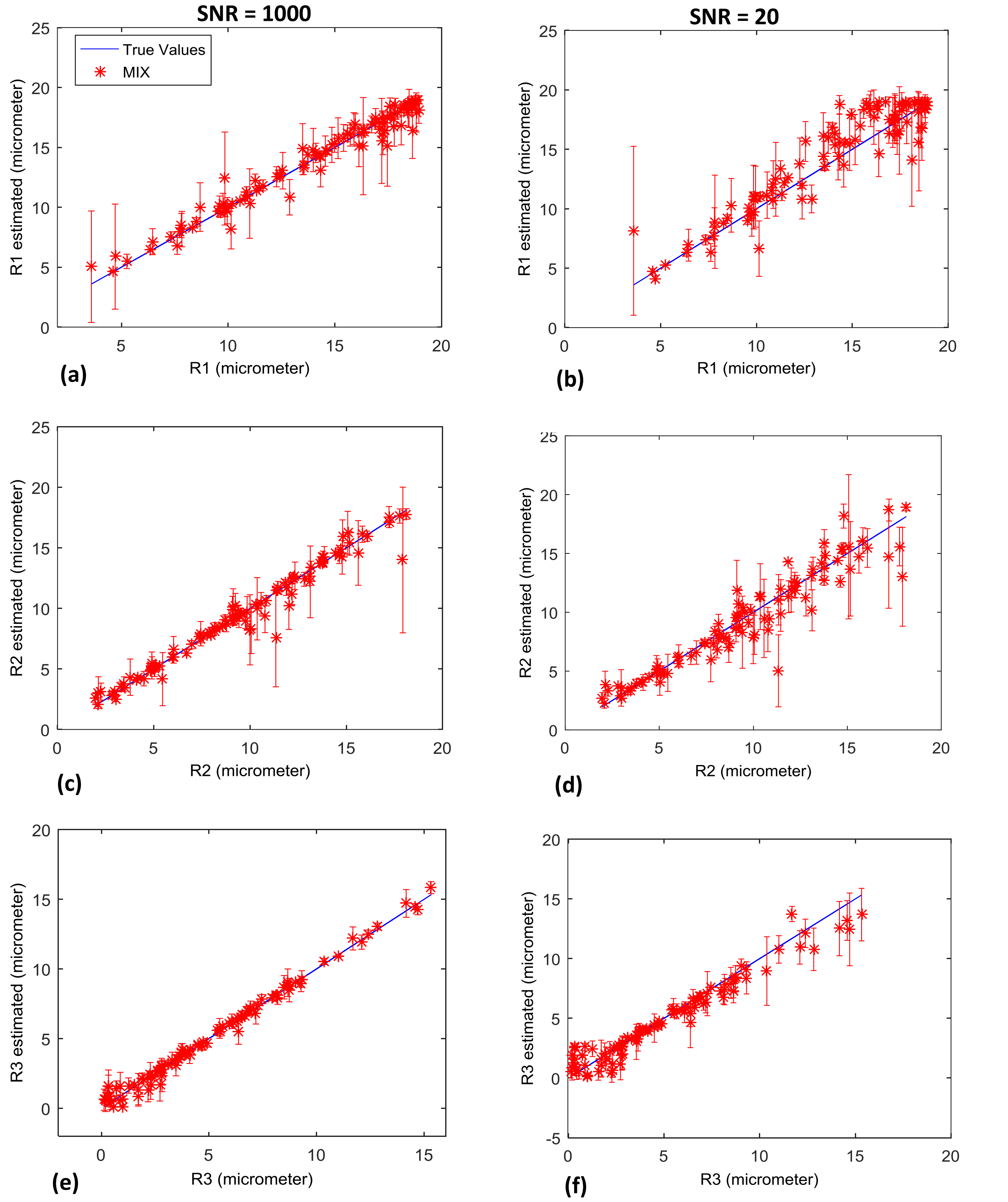

Supplementary Figure 4. Axon radius index estimates in three orientations using ZCDx model. R_1, R_2 and R_3 estimates are associated with the three different fiber orientations in a voxel. Results shown are 'errorbar' plots of 10 fitting results for both SNR= inf (left column) and SNR=20 (right column) data-sets. It can be seen that radius index can be estimated with good accuracy even at a reasonably low signal to noise ratio. With increased noise in the signal, there is a trend of over-estimating low radii and under-estimating larger radii.

Supplementary Figure 5: ZCDx parameters estimation using synthetic data (fiber orientation estimates)

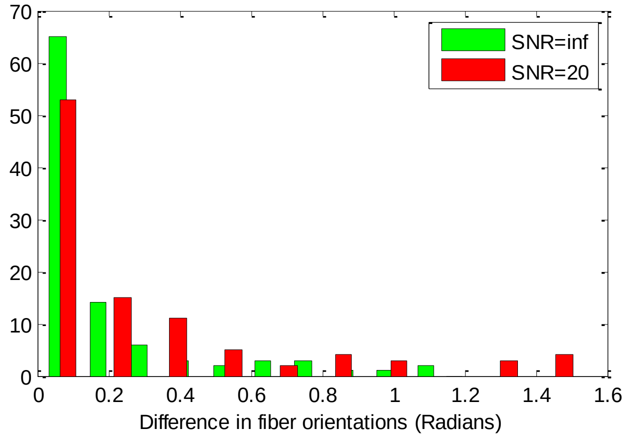

(a) Histogram of difference between actual and estimated first fiber orientation

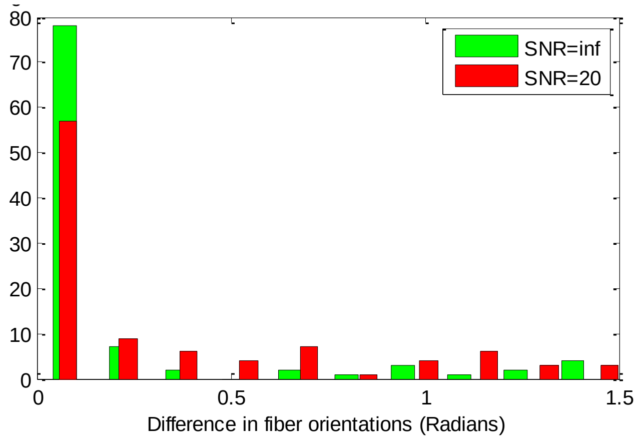

(b) Histogram of difference between actual and estimated second fiber orientation

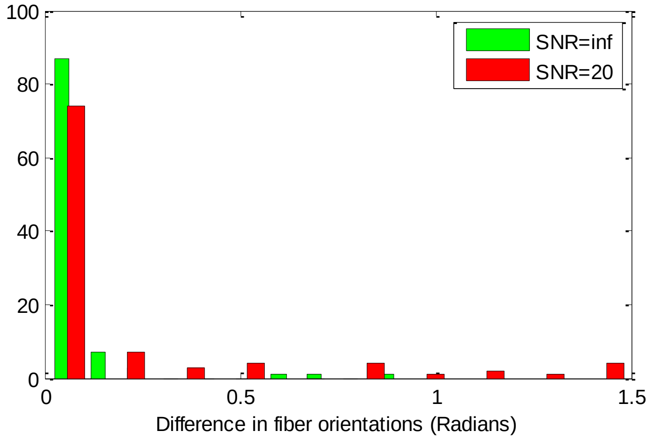

(c) Histogram of difference between actual and estimated third fiber orientation

Supplementary Figure 5. Fiber orientation estimation results in three orientations using ZCDx model.
